# A vascular endothelial growth factor receptor gene variant is associated with susceptibility to acute respiratory distress syndrome

**DOI:** 10.1186/s40635-018-0181-6

**Published:** 2018-07-09

**Authors:** Natalia Hernandez-Pacheco, Beatriz Guillen-Guio, Marialbert Acosta-Herrera, Maria Pino-Yanes, Almudena Corrales, Alfonso Ambrós, Leonor Nogales, Arturo Muriel, Elena González-Higueras, Francisco J. Diaz-Dominguez, Elizabeth Zavala, Javier Belda, Shwu-Fan Ma, Jesús Villar, Carlos Flores, the GEN-SEP Network, Jesús Villar, Rosa L. Fernández, Carlos Flores, Maria Pino-Yanes, Marialbert Acosta-Herrera, Lina Pérez-Méndez, Almudena Corrales, Elena Espinosa, David Domínguez, Jesús Blanco, Arturo Muriel, Victor Sagredo, Juan C. Ballesteros, Alfonso Ambrós, Rafael Cdel Campo, Francisco Gandía, Leonor Nogales, Rafael Fernández, Carles Subirá, Aurora Baluja, José M. Añón, Elena González, Rosario Solano, Demetrio Carriedo, Francisco J. Diaz-Dominguez, Ramón Adalia, Elizabeth Zavala

**Affiliations:** 1 Research Unit, Hospital Universitario N.S. de Candelaria, Universidad de La Laguna, Santa Cruz de Tenerife, Spain; 2 Research Unit, Hospital Universitario Dr. Negrin, Las Palmas de Gran Canaria, Spain; 3 CIBER de Enfermedades Respiratorias, Instituto de Salud Carlos III, Madrid, Spain; 4 Genomics and Health Group, Department of Biochemistry, Microbiology, Cell Biology and Genetics, Universidad de La Laguna, La Laguna, Tenerife Spain; 5 Intensive Care Unit, Hospital General de Ciudad Real, Ciudad Real, Spain; 6 Intensive Care Unit, Hospital Clínico Universitario de Valladolid, Valladolid, Spain; 7 Intensive Care Unit, Hospital Universitario Rio Hortega, Valladolid, Spain; 8 Intensive Care Unit, Hospital Virgen de La Luz, Cuenca, Spain; 9 Intensive Care Unit, Hospital General de León, León, Spain; 10 Intensive Care Unit, Hospital Clinic Barcelona, Barcelona, Spain; 11 Department of Anesthesiology, Hospital Clínico Universitario, Universidad de Valencia, Valencia, Spain; 12 Division of Pulmonary and Critical Care Medicine, Department of Medicine, University of Chicago, Chicago, USA; 13 Instituto de Parasitología y Biomedicina López-Neyra, IPBLN-CSIC, P.T.S, Granada, Spain

**Keywords:** Acute lung injury, Sepsis, Polymorphism, Genetic predisposition

## Abstract

**Background:**

The acute respiratory distress syndrome (ARDS) is one of the main causes of mortality in adults admitted to intensive care units. Previous studies have demonstrated the existence of genetic variants involved in the susceptibility and outcomes of this syndrome. We aimed to identify novel genes implicated in sepsis-induced ARDS susceptibility.

**Methods:**

We first performed a prioritization of candidate genes by integrating our own genomic data from a transcriptomic study in an animal model of ARDS and from the only published genome-wide association study of ARDS study in humans. Then, we selected single nucleotide polymorphisms (SNPs) from prioritized genes to conduct a case-control discovery association study in patients with sepsis-induced ARDS (*n* = 225) and population-based controls (*n* = 899). Finally, we validated our findings in an independent sample of 661 sepsis-induced ARDS cases and 234 at-risk controls.

**Results:**

Three candidate genes were prioritized: dynein cytoplasmic-2 heavy chain-1, fms-related tyrosine kinase 1 (*FLT1*), and integrin alpha-1. Of those, a SNP from *FLT1* gene (rs9513106) was associated with ARDS in the discovery study, with an odds ratio (OR) for the C allele of 0.76, 95% confidence interval (CI) 0.58–0.98 (*p* = 0.037). This result was replicated in an independent study (OR = 0.78, 95% CI = 0.62–0.98, *p* = 0.039), showing consistent direction of effects in a meta-analysis (OR = 0.77, 95% CI = 0.65–0.92, *p* = 0.003).

**Conclusions:**

We identified *FLT1* as a novel ARDS susceptibility gene and demonstrated that integration of genomic data can be a valid procedure to identify novel susceptibility genes. These results contribute to previous firm associations and functional evidences implicating *FLT1* gene in other complex traits that are mechanistically linked, through the key role of endothelium, to the pathophysiology of ARDS.

**Electronic supplementary material:**

The online version of this article (10.1186/s40635-018-0181-6) contains supplementary material, which is available to authorized users.

## Background

The acute respiratory distress syndrome (ARDS) is an acute and intense inflammatory process of the lung caused by injury to the alveolar-capillary membrane that results in increased permeability and protein-rich edema. Diagnosis is based on a constellation of clinical, radiographic, and physiologic abnormalities including severe hypoxemia, bilateral pulmonary infiltrates on chest-X-ray, and no clinical evidence of hydrostatic/cardiogenic pulmonary edema [1]. The estimated annual incidence of ARDS is 7 cases per 100,000 individuals, with an associated hospital mortality of 40% [2, 3]. Although there are different processes that can cause ARDS, such as pneumonia, severe trauma, or blood transfusions [4]; sepsis continues to be the main risk factor precipitating this syndrome in adults [5].

Several studies have demonstrated the influence of the individual’s genetic variation in the susceptibility to ARDS [6, 7]. It is suggested that such genetic factors not only could partially account for the heterogeneity of ARDS presentation and the variability in the response to therapies among patients, but also may explain the differential prevalence of ARDS among different populations [4, 8] and ethnic groups [2, 9–11]. A number of genes have been proposed as biological candidates for ARDS susceptibility based on gene expression studies in animal or cellular models followed by candidate gene association studies [6].

Despite the fruitful use of genome-wide association studies (GWAS) to unravel risk factors in many complex diseases, to date only one GWAS in patients with ARDS has been published and it was focused on trauma-induced ARDS. In that study, the protein tyrosine phosphatase, receptor type F-interacting protein alpha-1 (*PPFIA1*) gene was identified as a novel ARDS locus, which encodes a protein implicated in cell-cell junctions and cell interactions with the extracellular matrix [12]. The authors first identified the most significant association signals in the discovery stage, and then, a replication was performed in independent samples. Finally, these signals were recognized by integration with gene expression data [12]. However, it is anticipated that many other ARDS loci remain to be identified. In the present study, we aimed to reassess the GWAS results published by Christie et al. [12] performing the integration with transcriptomic data from an animal model of ARDS. This approach allowed to prioritize candidate genes that were followed-up in independent replication studies in order to reveal novel sepsis-induced ARDS susceptibility loci.

## Methods

### Prioritization of candidate genes

**Fig. 1:**
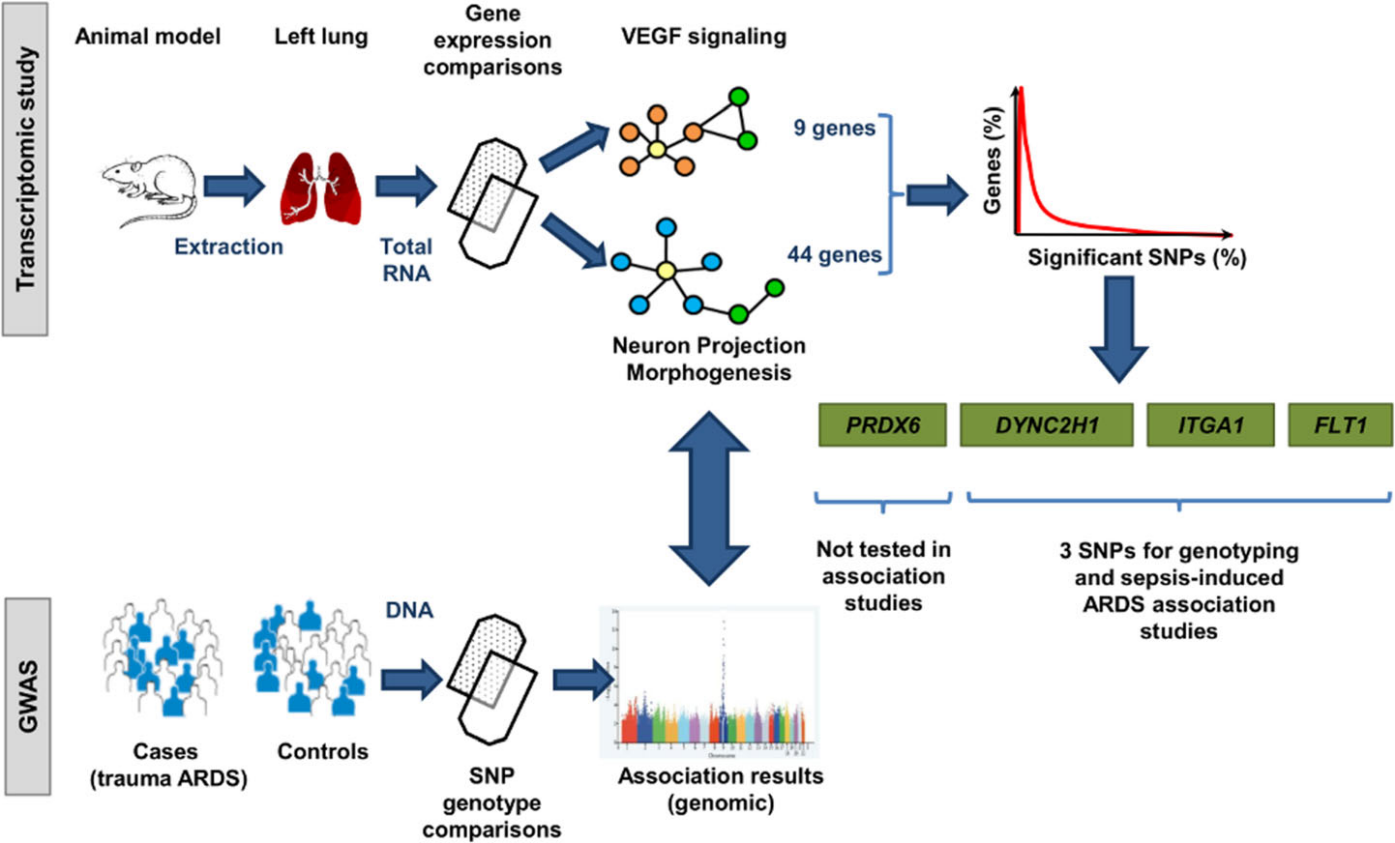
Schematic representation of study procedures for gene prioritization for case-control association studies with ARDS susceptibility. Total RNA from left lung tissue from surviving animals of a rodent model of ARDS was used to perform gene expression comparisons among three experimental groups on different mechanical ventilation (MV) strategies. These analyses revealed *neuron projection morphogenesis* and *signaling by vascular endothelial growth factor* (VEGF) as differentially deregulated pathways, as well as in independent human genomic data. Among the genes contained in both pathways (9 and 44 genes, respectively), only those with at least one significant SNP (*p* value ≤ 0.01) in the GWAS of ARDS were prioritized: dynein cytoplasmatic 2, heavy chain 1 (*DYNC2H1*), fms-related tyrosine kinase-1 (*FLT1*), integrin, alpha-1 (*ITGA1*), and peroxiredoxin-6 (*PRDX6*), the latter was excluded for association analyses because the top SNP was monomorphic in the discovery study

Figure 1 illustrates the overall scheme of the process involved in the selection of candidate genes. Gene prioritization began with transcriptomic data derived from an experimental animal model of ARDS, which was induced by abdominal sepsis after 24 h of cecal ligation and puncture, and subsequent challenge by mechanical ventilation (MV) strategies for 4 h [13] (ArrayExpress accession E-MTAB-2673). Three experimental groups were compared based on the ventilatory approach applied: unventilated spontaneously breathing septic animals (SS), septic animals ventilated with protective MV (SPV), and septic animals ventilated with injurious MV (SIV). Total RNA was purified from total left lung tissue of surviving animals from each experimental group, which was used for hybridization of fragmented cRNA to the GeneChip Rat Genome 230 2.0 Array (Affymetrix, Santa Clara, CA). Then, differential gene expression was identified using a multi-group comparison by means of *MulCom* test, revealing 2859 deregulated genes with a false discovery rate (FDR) ≤ 0.05 and fold change (FC) ≥ 1.7 (Additional file 1: Table S1). Differential contribution on biological processes of these genes was assessed by functional annotation clustering analyses using the Database for Annotation, Visualization and Integrated Discovery (DAVID) v6.7 [14]. This analysis revealed *Neuron projection morphogenesis* as the key deregulated pathway (FDR = 1.76 × 10^−3^) (Additional file 2: Table S2). Later, in order to reveal other common mechanisms in the animal model, we performed a protein-protein interaction network with the EnrichNet tool using the overlapping deregulated genes from the multi-group comparison (Additional file 3: Table S3). While eight signaling interaction networks were significant in at least one of the groups, the *signaling by vascular endothelial growth factor* (VEGF) network was the only significantly deregulated across all three groups. *Signaling by VEGF* plays a central and broad role in endothelial cell function, and in several aspects of the immune and inflammatory response, usually linked to lung edema promotion [15]. Moreover, *neuron projection morphogenesis* shares important characteristics with *signaling by VEGF*, such as the interplay of similar angiogenic molecules, principles, and mechanisms implicated in the navigation through their targets and morphogens [16]. In fact, VEGF could be considered a prototypic angioneurin, comparable to semaphorins, netrins, slits, ephrins, and neuropilins [17].

To validate these results, we then performed a post hoc evaluation from two independent public genomic studies of ARDS. The first was a transcriptomic study in blood cells from septic and sepsis-derived ARDS patients that had received MV for 48 h or less [18] (NCBI accession number GSE10474), which resembled the most experimental conditions from our animal model at that time (Additional file 4: Table S4). The second was the only genome-wide association study (GWAS) of ARDS published at the time [12] (10.1371/journal.pone.0028268.s003). In these two datasets, we observed that genes involved in neural and vascular functions were deregulated and nominally associated with ARDS.

The two pathways, *neuron projection morphogenesis* (Gene Ontology identifier GO:0048812) [19] and *signaling by VEGF* (KEGG identifier map04370) [20], contained 9 and 44 genes, respectively. These were followed up to prioritize the candidate genes for association studies. We then surveyed all SNPs in these 53 genes (including 100 kilobases (Kb) flanking sequences [21]) reported by Christie et al. [12] and retained the ones showing a *p* value ≤ 0.01. To take into account the multiple testing and to avoid potential prioritization bias due to gene length, we corrected the significance by the independent number of variants analyzed per gene, which was calculated with the SNPSpD software [22] based on the linkage disequilibrium (LD) information in the European population data from The 1000 Genomes Project [23].

Finally, either the most significant SNP or a SNP in strong LD among those genes surviving multiple testing corrections was put forward for association studies. LD patterns were obtained with Haploview in the form of pairwise *r*^*2*^ values [24].

### Association study: discovery

A case-control study was performed in DNA samples from unrelated Spanish individuals. As cases, we studied 429 patients with severe sepsis admitted into a network of intensive care units (ICUs) in Spain (see “Acknowledgements”) who were followed for the development of ARDS (*n* = 230), according to the definition of the American-European Consensus Criteria [1] and as having moderate or severe ARDS according to the Berlin criteria [25]. All patients were managed with a lung-protective MV strategy using low tidal volume (4–8 ml/kg predicted body weight) and PEEP ≥ 5 cmH_2_O at the time of meeting ARDS definition. Controls were derived from a population-based study with nationwide recruitment by the Spanish National DNA Biobank [26] and consisted of 900 individuals self-reporting of having four grandparents of Spanish origin and with no history of respiratory disease or major infections. DNA was extracted using the GFX kit (GE Healthcare, Little Chalfont, UK) following the manufacturer’s recommendations. Genotyping of the SNPs selected was performed with KASP™ probes (LGC Genomics, Teddington, UK) using a 7500 Fast Real-Time PCR System (Life Technologies, Carlsbad, CA). Genotype calls were automatically assigned by discriminating analysis with 95% confidence using the SDS software (Life Technologies). A total of 60 samples were genotyped by duplicate to monitor the genotyping reproducibility by measuring the concordance rate. SNP genotyping quality control and deviations from the Hardy-Weinberg equilibrium (HWE) were assessed with R 3.2.2 [27]. SNP minor allele frequency (MAF) in the control group was calculated and compared to the values reported for the Iberian Population in Spain from The 1000 Genomes Project to assess its consistency [23].

**Fig. 2:**
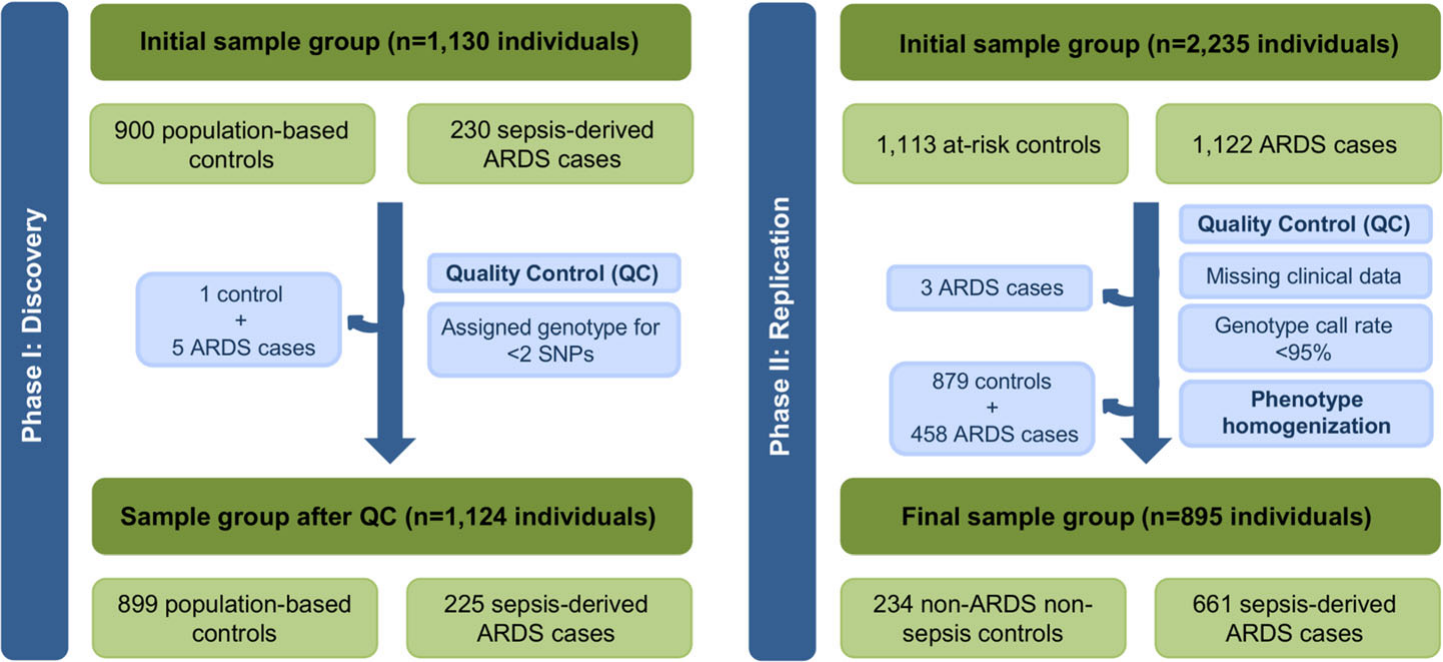
Workflow of the study design. Quality control steps performed in each stage for the samples analyzed. Abbreviations: QC quality control, ARDS acute respiratory distress syndrome

We performed association analyses of the individual SNPs with ARDS susceptibility using logistic regressions models in R, comparing ARDS cases and population-based controls that were retained after quality controls (Fig. 2 and Table 1).

**Table 1:** Characteristics of ARDS patients and controls of the discovery study

Characteristics	Cases (*n* = 225)	Controls (*n* = 889)
Gender (% male)	37	41
Mean age (years) (*P*_25_–*P*_75_)	63 (54–75)	41 (32–49)
Hypertension (%)	44	3
Smoker (%)	30	49
Previous surgery (%)	53	NA
Ischemic cardiac disease (%)	11	NA
Source of sepsis (%)		
Pulmonary	49	NA
Extrapulmonary	51	NA
Pathogen (%)		
Gram-negative	49	NA
Gram-positive	33	NA
Gram-negative and Gram-positive	5	NA
Polymicrobial	2	NA
Virus	6	NA
Fungi	3	NA
Organ dysfunction (%)		
Circulatory	62	NA
Renal	42	NA
Hepatic	18	NA
Neurologic	22	NA
Coagulation	22	NA
APACHE II (median) (*P*_25_–*P*_75_)	22 (18–27)	NA
ICU mortality (%)	44	NA

*APACHE II* Acute Physiology and Chronic Health Evaluation II, *ARDS* acute respiratory distress syndrome, *ICU* intensive care unit, *P*_*25*_ percentile 25, *P*_*75*_ percentile 75, *NA* not available

### Association study: replication

Replication was assessed in an independent sample set obtained from the database of Genotypes and Phenotypes (dbGaP) from a GWAS by the ARDSnet and the iSPAAR Consortium (study accession phs000631.v1.p1). According to the information deposited in the dbGaP database, a total of 518,313 SNPs were genotyped using the Illumina genotyping platform Human660W-Quad v1.0 in 2235 Western European descent individuals, including 1122 ARDS cases and 1113 at-risk controls (i.e., patients with pneumonia, trauma, and pancreatitis or those subjected to aspiration or massive blood transfusion but did not develop ARDS). We performed quality control procedures with PLINK v1.07 [28] to remove samples with missing clinical information and genotype call rates < 95%, leaving a total of 1119 cases and 1113 controls for further analyses. Additionally, those SNPs with a genotyping rate < 97% were excluded, leaving a total of 516,348 SNPs, none of which deviated from HWE (*p* value < 1 × 10^−6^). In addition, principal component analysis was performed with EIGENSOFT (v4.2) [29] analyzing ~ 100,000 independent SNPs extracted from this data. In order to retrieve the SNPs of interest, genotype imputation was performed with Minimac3 [30] using the Haplotype Reference Consortium version r1.1 data as reference panel [31] on the Michigan Imputation Server [32]. Association testing was conducted using logistic regression models with R including the first two principal components as covariates, showing a minimal inflation due to population stratification (Lambda = 1.036). To approximate as much as possible this study to that utilized in the discovery stage, the replication only considered as cases those patients with sepsis-associated ARDS (*n* = 661) and as controls those patients that were free from sepsis and ARDS (*n* = 234) (Fig. 2). Detailed demographic and clinical data from the subset of patients included in this stage can be found in Table 2.

**Table 2:** Characteristics of ARDS patients and controls of the replication study

Characteristics	Complete dataset (*n* = 2235)^a^		Analyzed dataset (*n* = 895)^b^	
	Cases (*n* = 1122)	Controls (*n* = 1113)	Cases (*n* = 661)	Controls (*n* = 234)
Gender (% male)	56	61	58	67
Mean age (years) (*P*_25_–*P*_75_)	56 (44–70)	62 (51–75)	60 (48–73)	58 (45–73)
Ventilator-free days (median) (*P*_25_–*P*_75_)	17 (0–24)	27 (24–29)	14 (0–23)	27 (24–28)
Mortality (%)	23	6	28	5

*ARDS* acute respiratory distress syndrome, *P*_*25*_ percentile 25, *P*_*75*_ percentile 75

^a^Total number of individuals included in the GWAS study by the ARDSnet and the iSPAAR Consortium (ARDS cases and at-risk controls) according to dbGaP study phs000631.v1.p1

^b^Subset of individuals selected to perform the association study (septic-induced ARDS cases and non-septic and non-ARDS controls)

### Meta-analysis of associations and evaluation of functionality

A meta-analysis was performed with the METASOFT software [33] to estimate the overall effect size of associated SNPs across the two studies (discovery and replication cohorts) and to evidence the existence of heterogeneity of effects using Cochran’s *Q* statistic *p* value. Statistical power to detect effects larger that an odds ratio (OR) of 1.35 for a MAF = 22% at 0.01 significance threshold was estimated as 93.3% (http://osse.bii.a-star.edu.sg/index.php).

To assess the functional role of the SNPs, the online tool Haploreg v4.1 [34] was used to query the empirical data provided by the Encyclopedia of DNA Elements (ENCODE) project. Moreover, updated information related to expression Quantitative Trait Loci (eQTL) were inspected for the SNPs selected for each gene included in the association analyses or any SNP in large LD (*r*^*2*^ ≥ 0.8). For that, several publicly available datasets were explored, including the GTEx Portal [35] and SiNoPsis v1.0 (https://compgen.bio.ub.edu/SiNoPsis/).

## Results and discussion

Thirteen out of 53 genes from the *signaling by VEGF* and *neuron projection morphogenesis* pathways showed at least one SNP associated with trauma-induced ARDS in the GWAS data at the nominal significance level reported in the published study (*p* ≤ 0.01) (Fig. 1; Additional file 5: Table S5). After correcting for the independent number of SNPs analyzed in each gene, four genes contained the largest proportion of significant SNPs normalized by number of variants analyzed per gene, namely dynein cytoplasmatic 2, heavy chain 1 (*DYNC2H1*), fms-related tyrosine kinase-1 (*FLT1*), integrin, alpha-1 (*ITGA1*), and peroxiredoxin-6 (*PRDX6*). For each of them, the most significant SNP, or a SNP in high LD with it, was chosen for genotyping. For *DYNC2H1* and *ITGA1*, we selected the SNPs rs11225640 (*p* = 3.5 × 10^−5^ with trauma-induced ARDS) and rs16880534 (*p* = 4.7 × 10^−3^ with trauma-induced ARDS), respectively. However, for the *FLT1* gene, the flanking region of the top-prioritized SNP (rs9508026, *p* = 1.1 × 10^−3^ with trauma-induced ARDS) did not allow the design of a genotyping assay. Therefore, this top-associated SNP was captured by an alternative SNP (rs9513106) in strong LD (*r*^*2*^ = 0.8). Lastly, for *PRDX6*, we selected the variant rs9425722 (*p* = 3.2 × 10^−3^ with trauma-induced ARDS).

In the discovery study, association testing was performed in 1124 individuals (225 sepsis-induced ARDS cases and 899 controls) after removing six subjects (1 control and 5 cases) whose genotype could not be determined for ≥ 2 SNPs (Fig. 2). Among cases, 49% had a positive blood culture, where Gram-negative bacteria were the causing agents of sepsis. Additionally, the most likely origin of sepsis was pulmonary (49% of patients), and the overall ICU mortality rate was 44% (Table 1).

The *PRDX6* variant selected (rs9425722) resulted monomorphic in our samples (results not shown). This result was consistent with the information found in the European population data from The 1000 Genomes Project [23], where this position is monomorphic. For this reason, and given that a previous study evaluated the common variants from the *PRDX6* gene and found no association with ARDS [36], we disregarded this gene from further analyses. The other three SNPs selected for association studies had a genotype concordance rate > 98% among duplicated samples and a genotyping rate > 95% (Table 3), and none of the SNPs deviated significantly from the HWE in the population-based controls. Association testing of these three SNPs with the development of ARDS susceptibility (Additional file 6: Table 4) revealed a significant association for the *FLT1* SNP (rs9513106), with an OR for the C allele of 0.76 (95% confidence interval (CI) = 0.58–0.98, *p* = 0.037). This protective effect of the C allele was validated in the replication study (661 sepsis-associated ARDS cases and 234 controls) showing an OR for the C allele of 0.78 (95% CI = 0.62–0.98) and a *p* = 0.039. No heterogeneity of effects was found between the two studies (Cochran’s Q, *p* = 0.850) and concordance in the direction of effects was confirmed in a fixed-effect meta-analysis comprising a total of 886 sepsis-induced ARDS cases and 1133 controls (OR = 0.77, 95% CI = 0.65–0.92, *p* = 0.003).

**Table 3:** Summary metrics and allelic frequencies of genotyped SNPs in the discovery study

SNP	Gene	Minor allele	Concordance rate (95% CI)	Completion rate (%)	HWE^a^	MAF^b^	
						Cases	Controls
rs9513106	*FLT1*	C	100 (95.7–100)	95.0	0.213	0.221	0.270
rs11225640	*DYNC2H1*	C	98.9 (94.1–99.8)	97.9	0.428	0.103	0.116
rs16880534	*ITGA1*	G	100 (95.9–100)	99.6	0.117	0.216	0.199

^a^Pearson’s χ^2^ test

^b^Minor allele frequency

The *FLT1* gene encodes a tyrosine kinase receptor with an extracellular ligand-binding region with seven domains of immunoglobulin that belongs to the VEGF receptor family. Ligands of FLT1, such as VEGF-A, VEGF-B, and placental growth factors are critically involved in edema formation during ARDS [15]. After binding FLT1, they trigger intracellular signaling pathways involved in cellular proliferation, survival, and migration, as well as in the increase of endothelial cell permeability and angiogenesis [37]. Studies carried out in animal models support the implication of this pathway promoting vascular permeability [38], and functional assays in human vascular cells have shown that elevated expression of *FLT1* gene was associated with decreased amounts of inflammatory adhesion molecules [39]. VEGF signaling is required for the maintenance of adult lung alveolar structures and reduced VEGF levels have been found in bronchoalveolar lavage (BAL) samples from ARDS patients [40–42], in contrast with the high VEGF levels found in BAL from healthy individuals [43]. Furthermore, oxidative stress, cytokines, and hypoxia are important factors that regulate VEGF expression [44], which are key players in ARDS development. Therefore, a positive feedback may exist between all or some of these factors to increase VEGF expression, and consequently, VEGF binding to its receptors may contribute to the promotion of increased vascular permeability and a higher degree of hypoxemia as it has been reported in the earliest stages of ARDS [45]. Common variants in VEGF signaling genes have been consistently associated with susceptibility or outcomes of ARDS patients, and *VEGF* is one of the most replicated genes associated with ARDS [6]. However, to date, variants from *FLT1* have never been associated with ARDS susceptibility or outcomes, although some of them have been found to be associated with other relevant complex traits. In this sense, the *FLT1* variant identified in this study was previously associated in two GWAS of large sample sizes and with multi-ethnic patients with coronary arterial disease [46, 47], where endothelium also plays a key pathophysiological role. Besides, a recent study has shown that two *FLT1* variants are associated with a reduced count of red blood cells [48], and with risk of preeclampsia, perhaps influencing the maternal endothelial dysfunction [48, 49]. Taken together, this information supports the firm role of *FLT1* gene variants in complex traits that are mechanistically linked, through the key role of endothelium, to the pathophysiology of the ARDS.

Our findings reinforce the critical importance of variants in genes from the VEGF pathway in ARDS susceptibility. Despite the fact that the *FLT1* SNP rs9513106 is located in an intronic region, the nucleotide change predicts alterations in two transcription regulatory motifs for the members binding to the GATA DNA elements and signal transducer and activator of transcription (STAT) factor families according to Haploreg v4.1. However, no significant eQTL results were found for rs9513106 or any SNP in strong LD in Europeans. In addition, as demonstrated by experiments performed as part of the ENCODE project, rs9513106 is in strong LD with another intronic SNP 9.6 Kb upstream, rs9508025. This SNP is located in a highly conserved region among species, in particular, in an enhancer histone mark and a DNAse I hypersensitivity site in fetal lung cells. Interestingly, rs9508025 was identified as one of the top-hits associated with coronary artery disease risk in a large GWAS study [50]. Nevertheless, future studies are needed to assess the functional effects of these variants in *FLT1* expression, protein activity, or its relation with the soluble forms of the protein (i.e., sFLT1) in patient samples. It should be mentioned that increased levels of sFLT1 in plasma have been related with sepsis severity, organ dysfunction, and mortality [51, 52]. Additionally, high sFLT1 levels have also been found in BAL samples of ARDS patients [53]. Therefore, given that sFLT1 acts as a natural competitive inhibitor of VEGF [54], we postulate that the protective genetic variant found in *FLT1* (or a variant in LD with it) could promote the increase of sFLT1 levels and hence, contributing to the protection from lung injury by reducing VEGF activity and the vascular permeability in ARDS patients. Further studies will be needed to evaluate this possibility.

The main strength of our study is that we found an association of the *FLT1* SNP rs9513106 with sepsis progression towards ARDS in the discovery sample using population-based controls and that this SNP was consistently replicated in an independent sample using hospitalized at-risk controls. Therefore, the effect of environmental factors related with the hospital setting or study design on the association of the *FLT1* variant with ARDS susceptibility is minimal. In addition, since no adjustment for population stratification was performed in the discovery sample, the replication in an independent sample with appropriate adjustments suggests minimal confounding effects due to population stratification. However, the top prioritized finding in the published GWAS of ARDS (rs9508026) [12] was not tested due to the fact that this did not allow the design of a genotyping assay so that, an alternative SNP was captured. In addition, although we prioritized the top significant SNP, we only considered one variant per gene for association studies. Therefore, other genetic variants not analyzed in our study (including rarer variants) could also be relevant in this syndrome, and this limitation could explain the lack of significance in the results for the other three genes.

Regarding the control dataset used in the discovery phase, population-based controls are considered the most obvious control group, being a random sample from the source population where cases were obtained [55]. The use of this kind of controls allows reducing the possible selection bias caused by differences among cases and controls beyond the disease process itself and potential risk factors [56, 57]. Additionally, they provide stronger association effect estimates than hospital-based controls when cases have been recruited from hospitals since the association evidence revealed by population-based controls include those obtained by using controls from the hospital [56]. As a drawback, potential confounders related to the differences in age and comorbidities among cases and the population-based controls may have affected the findings of the discovery study. However, the results replicated in the independent ARDSnet/iSPAAR Consortium study, where no differences in age and comorbidities were detected (controls were patients at-risk) allowing to control more closely the environmental factors. This strongly reinforces the validity of our findings.

We propose that the prioritization of genes based on pathway overlapping among distinct data sources (animal and human) and studies (transcriptomics and GWAS) would provide firm deregulated mechanisms during the acute lung injury, leading to the selection of more robust genes, contrasting to classical association studies that focus on a biological candidate based on literature information. For now, our studies preclude concluding whether or not the *FLT1* gene variant is directly associated with sepsis-induced ARDS susceptibility or with the aggravation of sepsis. However, whatever the case, it is equally valid and relevant information. Further studies would be needed to disentangle the main driver of such association.

## Conclusions

In conclusion, here we report for the first time the association of a common variant in the *FTL1* gene with sepsis-induced ARDS susceptibility. Our findings demonstrate that the integration of information from different sources of public genomic data could be an efficient method to identify novel genes for ARDS susceptibility.

## Additional files

Additional file 1: **Table S1.** Significant probe sets in the microarray-based differential gene expression analysis comparing experimental sepsis groups with a common control group (data obtained from Acosta-Herrera et al. PLoS One 2015, 10:e0132296). Description of data: statistics obtained from the pathway enrichment analysis performed for each experimental group included in the transcriptomic lung study before the prioritization of candidate genes. (XLS 431 kb)

Additional file 2: **Table S2.** Pathway enrichment analysis performed in each experimental septic group. Description of data: summary results of the pathway enrichment analysis. (DOC 51 kb)

Additional file 3: **Table S3.** Protein interaction network analysis (data obtained from Acosta-Herrera et al. PLoS One 2015, 10:e0132296). Summary results of the protein-protein interaction network analysis as part of the lung transcriptomic study previously published. (DOC 40 kb)

Additional file 4: **Table S4.** Summary results of the transcriptomic study in blood cells from septic and sepsis-derived ARDS patients. Summary results of the validation from GSEA in whole blood performed in a transcriptomic study. (DOC 31 kb)

Additional file 5: **Table S5.** Summary statistics of SNPs located at the pathways with SNPs significantly associated with ARDS by Christie et al. (PLoS One 2012, 7:e28268). Summary results of the prioritization performed from the 13 genes with significant SNPs at the GWAs of ARDS performed by Christie et al. (XLS 45 kb)

Additional file 6: **Table 4.** ARDS susceptibility association results in the discovery and replication studies. Summary of association results for the three SNPs associated with ARDS susceptibility in the discovery and replication studies. (DOC 40 kb)

## Abbreviations

ARDS: Acute respiratory distress syndrome
BAL: Bronchoalveolar lavage
CI: Confidence interval
dbGaP: Database of Genotypes and Phenotypes
*DYNC2H1*: Dynein cytoplasmic-2 heavy chain-1
ENCODE: Encyclopedia of DNA Elements
eQTL: Expression Quantitative Trait Loci
FC: Fold change
FDR: False discovery rate
*FLT1*: Fms-related tyrosine kinase 1
GWAS: Genome-wide association studies
HWE: Hardy-Weinberg equilibrium
ICU: Intensive care unit
*ITGA1*: Integrin, alpha-1
LD: Linkage disequilibrium
MAF: Minor allele frequency
MV: Mechanical ventilation
OR: Odds ratio
PEEP: Positive end-expiratory pressure
*PPFIA1*: Protein Tyrosine Phosphatase, Receptor Type F-interacting protein alpha 1
*PRDX6*: Peroxiredoxin-6
SIV: Septic animals ventilated with injurious mechanical ventilation
SNPs: Single nucleotide polymorphisms
SPV: Septic animals ventilated with protective mechanical ventilation
SS: Unventilated spontaneously breathing septic animals
STAT: Signal transducer and activator of transcription
VEGF: Vascular endothelial growth factor

## References

[1] Bernard GR, Artigas A, Brigham KL, Carlet J, Falke K, Hudson L, Lamy M, Legall JR, Morris A, Spragg R (1994). The American-European Consensus Conference on ARDS. Definitions, mechanisms, relevant outcomes, and clinical trial coordination. Am J Respir Crit Care Med.

[2] Villar J, Blanco J, Anon JM, Santos-Bouza A, Blanch L, Ambros A, Gandia F, Carriedo D, Mosteiro F, Basaldua S (2011). The ALIEN study: incidence and outcome of acute respiratory distress syndrome in the era of lung protective ventilation. Intensive Care Med.

[3] Villar J, Blanco J, Kacmarek RM (2016). Current incidence and outcome of the acute respiratory distress syndrome. Curr Opin Crit Care.

[4] Rubenfeld GD, Caldwell E, Peabody E, Weaver J, Martin DP, Neff M, Stern EJ, Hudson LD (2005). Incidence and outcomes of acute lung injury. N Engl J Med.

[5] Matthay MA, Zimmerman GA (2005). Acute lung injury and the acute respiratory distress syndrome: four decades of inquiry into pathogenesis and rational management. Am J Respir Cell Mol Biol.

[6] Acosta-Herrera M, Pino-Yanes M, Perez-Mendez L, Villar J, Flores C (2014). Assessing the quality of studies supporting genetic susceptibility and outcomes of ARDS. Front Genet.

[7] Flores C, Pino-Yanes Mdel M, Villar J (2008). A quality assessment of genetic association studies supporting susceptibility and outcome in acute lung injury. Crit Care.

[8] Villar J, Perez-Mendez L, Blanco J, Anon JM, Blanch L, Belda J, Santos-Bouza A, Fernandez RL, Kacmarek RM (2013). A universal definition of ARDS: the PaO2/FiO2 ratio under a standard ventilatory setting—a prospective, multicenter validation study. Intensive Care Med.

[9] Martin GS, Mannino DM, Eaton S, Moss M (2003). The epidemiology of sepsis in the United States from 1979 through 2000. N Engl J Med.

[10] Erickson SE, Shlipak MG, Martin GS, Wheeler AP, Ancukiewicz M, Matthay MA, Eisner MD (2009). Racial and ethnic disparities in mortality from acute lung injury. Crit Care Med.

[11] Linko R, Okkonen M, Pettila V, Perttila J, Parviainen I, Ruokonen E, Tenhunen J, Ala-Kokko T, Varpula T (2009). Acute respiratory failure in intensive care units. FINNALI: a prospective cohort study. Intensive Care Med.

[12] Christie JD, Wurfel MM, Feng R, O'Keefe GE, Bradfield J, Ware LB, Christiani DC, Calfee CS, Cohen MJ, Matthay M (2012). Genome wide association identifies PPFIA1 as a candidate gene for acute lung injury risk following major trauma. PLoS One.

[13] Acosta-Herrera M, Lorenzo-Diaz F, Pino-Yanes M, Corrales A, Valladares F, Klassert TE, Valladares B, Slevogt H, Ma SF, Villar J, Flores C (2015). Lung transcriptomics during protective ventilatory support in sepsis-induced acute lung injury. PLoS One.

[14] Dennis G, Sherman BT, Hosack DA, Yang J, Gao W, Lane HC (2003). Lempicki RA (2003) DAVID: database for annotation, visualization, and integrated discovery. Genome Biol.

[15] Mura M, dos Santos CC, Stewart D, Liu M (2004). Vascular endothelial growth factor and related molecules in acute lung injury. J Appl Physiol.

[16] Larrivee B, Freitas C, Suchting S, Brunet I, Eichmann A (2009). Guidance of vascular development: lessons from the nervous system. Circ Res.

[17] Segura I, De Smet F, Hohensinner PJ, Ruiz de Almodovar C, Carmeliet P (2009). The neurovascular link in health and disease: an update. Trends Mol Med.

[18] Howrylak JA, Dolinay T, Lucht L, Wang Z, Christiani DC, Sethi JM, Xing EP, Donahoe MP, Choi AM (2009). Discovery of the gene signature for acute lung injury in patients with sepsis. Physiol Genomics.

[19] The Gene Ontology Consortium (2017). Expansion of the Gene Ontology knowledgebase and resources. Nucleic Acids Res.

[20] Kanehisa M, Goto S (2000). KEGG: kyoto encyclopedia of genes and genomes. Nucleic Acids Res.

[21] Cookson W, Liang L, Abecasis G, Moffatt M, Lathrop M (2009). Mapping complex disease traits with global gene expression. Nat Rev Genet.

[22] Nyholt DR (2004). A simple correction for multiple testing for single-nucleotide polymorphisms in linkage disequilibrium with each other. Am J Hum Genet.

[23] Abecasis GR, Auton A, Brooks LD, DePristo MA, Durbin RM, Handsaker RE, Kang HM, Marth GT, McVean GA (2012). An integrated map of genetic variation from 1,092 human genomes. Nature.

[24] Barrett JC, Fry B, Maller J, Daly MJ (2005). Haploview: analysis and visualization of LD and haplotype maps. Bioinformatics.

[25] Ranieri VM, Rubenfeld GD, Thompson BT, Ferguson ND, Caldwell E, Fan E, Camporota L, Slutsky AS, ARDS Definition Task Force (2012). Acute respiratory distress syndrome: the Berlin definition. JAMA.

[26] Spanish National DNA Biobank, Salamanca. http://www.bancoadn.org. Accessed 10 Sept 2014

[27] R Development Core Team (2008) R: a language and environment for statistical computing, Viena, Austria. http://www.R-project.org

[28] Purcell S, Neale B, Todd-Brown K, Thomas L, Ferreira MA, Bender D, Maller J, Sklar P, de Bakker PI, Daly MJ, Sham PC (2007). PLINK: a tool set for whole-genome association and population-based linkage analyses. Am J Hum Genet.

[29] Price AL, Patterson NJ, Plenge RM, Weinblatt ME, Shadick NA, Reich D (2006). Principal components analysis corrects for stratification in genome-wide association studies. Nat Genet.

[30] Fuchsberger C, Abecasis GR, Hinds DA (2015). minimac2: faster genotype imputation. Bioinformatics.

[31] McCarthy S, Das S, Kretzschmar W, Delaneau O, Wood AR, Teumer A, Kang HM, Fuchsberger C, Danecek P, Sharp K (2016). A reference panel of 64,976 haplotypes for genotype imputation. Nat Genet.

[32] Das S, Forer L, Schonherr S, Sidore C, Locke AE, Kwong A, Vrieze SI, Chew EY, Levy S, McGue M (2016). Next-generation genotype imputation service and methods. Nat Genet.

[33] Han B, Eskin E (2011). Random-effects model aimed at discovering associations in meta-analysis of genome-wide association studies. Am J Hum Genet.

[34] Ward LD, Kellis M (2012). HaploReg: a resource for exploring chromatin states, conservation, and regulatory motif alterations within sets of genetically linked variants. Nucleic Acids Res.

[35] GTEx Consortium (2013). The genotype-tissue expression (GTEx) project. Nat Genet.

[36] Rushefski M, Aplenc R, Meyer N, Li M, Feng R, Lanken PN, Gallop R, Bellamy S, Localio AR, Feinstein SI (2011). Novel variants in the PRDX6 gene and the risk of acute lung injury following major trauma. BMC Med Genet.

[37] Matsumoto T, Mugishima H (2006). Signal transduction via vascular endothelial growth factor (VEGF) receptors and their roles in atherogenesis. J Atheroscler Thromb.

[38] Hamada N, Kuwano K, Yamada M, Hagimoto N, Hiasa K, Egashira K, Nakashima N, Maeyama T, Yoshimi M, Nakanishi Y (2005). Anti-vascular endothelial growth factor gene therapy attenuates lung injury and fibrosis in mice. J Immunol.

[39] Konta A, Ozaki K, Sakata Y, Takahashi A, Morizono T, Suna S, Onouchi Y, Tsunoda T, Kubo M, Komuro I (2016). A functional SNP in FLT1 increases risk of coronary artery disease in a Japanese population. J Hum Genet.

[40] Maitre B, Boussat S, Jean D, Gouge M, Brochard L, Housset B, Adnot S, Delclaux C (2001). Vascular endothelial growth factor synthesis in the acute phase of experimental and clinical lung injury. Eur Respir J.

[41] Thickett DR, Armstrong L, Millar AB (2002). A role for vascular endothelial growth factor in acute and resolving lung injury. Am J Respir Crit Care Med.

[42] Abadie Y, Bregeon F, Papazian L, Lange F, Chailley-Heu B, Thomas P, Duvaldestin P, Adnot S, Maitre B, Delclaux C (2005). Decreased VEGF concentration in lung tissue and vascular injury during ARDS. Eur Respir J.

[43] Kaner RJ, Crystal RG (2001). Compartmentalization of vascular endothelial growth factor to the epithelial surface of the human lung. Mol Med.

[44] Ferrara N, Gerber HP, LeCouter J (2003). The biology of VEGF and its receptors. Nat Med.

[45] Kaner RJ, Ladetto JV, Singh R, Fukuda N, Matthay MA, Crystal RG (2000). Lung overexpression of the vascular endothelial growth factor gene induces pulmonary edema. Am J Respir Cell Mol Biol.

[46] Deloukas P, Kanoni S, Willenborg C, Farrall M, Assimes TL, Thompson JR, Ingelsson E, Saleheen D, Erdmann J, Goldstein BA (2013). Large-scale association analysis identifies new risk loci for coronary artery disease. Nat Genet.

[47] Howson JMM, Zhao W, Barnes DR, Ho WK, Young R, Paul DS, Waite LL, Freitag DF, Fauman EB, Salfati EL (2017). Fifteen new risk loci for coronary artery disease highlight arterial-wall-specific mechanisms. Nat Genet.

[48] McGinnis R, Steinthorsdottir V, Williams NO, Thorleifsson G, Shooter S, Hjartardottir S, Bumpstead S, Stefansdottir L, Hildyard L, Sigurdsson JK (2017). Variants in the fetal genome near FLT1 are associated with risk of preeclampsia. Nat Genet.

[49] Maynard SE, Min JY, Merchan J, Lim KH, Li J, Mondal S, Libermann TA, Morgan JP, Sellke FW, Stillman IE (2003). Excess placental soluble fms-like tyrosine kinase 1 (sFlt1) may contribute to endothelial dysfunction, hypertension, and proteinuria in preeclampsia. J Clin Invest.

[50] Lee JY, Lee BS, Shin DJ, Woo Park K, Shin YA, Joong Kim K, Heo L, Young Lee J, Kyoung Kim Y, Jin Kim Y (2013). A genome-wide association study of a coronary artery disease risk variant. J Hum Genet.

[51] Shapiro NI, Schuetz P, Yano K, Sorasaki M, Parikh SM, Jones AE, Trzeciak S, Ngo L, Aird WC (2010). The association of endothelial cell signaling, severity of illness, and organ dysfunction in sepsis. Crit Care.

[52] Skibsted S, Jones AE, Puskarich MA, Arnold R, Sherwin R, Trzeciak S, Schuetz P, Aird WC, Shapiro NI (2013). Biomarkers of endothelial cell activation in early sepsis. Shock.

[53] Perkins GD, Roberts J, McAuley DF, Armstrong L, Millar A, Gao F, Thickett DR (2005). Regulation of vascular endothelial growth factor bioactivity in patients with acute lung injury. Thorax.

[54] Kendall RL, Thomas KA (1993). Inhibition of vascular endothelial cell growth factor activity by an endogenously encoded soluble receptor. Proc Natl Acad Sci U S A.

[55] Thomas D (2006) Statistical methods in genetic epidemiology. Int J Epidemiol. USA, Oxford University Press; p. 501

[56] Neupane B, Walter SD, Krueger P, Loeb M (2010). Community controls were preferred to hospital controls in a case-control study where the cases are derived from the hospital. J Clin Epidemiol.

[57] Weiss ST (2001). Association studies in asthma genetics. Am J Respir Crit Care Med.

