# Supplementary material for: A vascular endothelial growth factor receptor gene variant is associated with susceptibility to acute respiratory distress syndrome
Source: Intensive Care Med Exp. 2018 Jul 9;6:16. doi: 10.1186/s40635-018-0181-6 (PMC6037659; doi:10.1186/s40635-018-0181-6)
Supplement: Supplementary file 2 — Table S2. Pathway enrichment analysis performed in each experimental septic group. Description of data: summary results of the pathway enrichment analysis. (DOC 51 kb) [file 40635_2018_181_MOESM2_ESM.doc]

| **Table S2. Pathway enrichment analysis performed in each experimental septic group.** | | | | | | | | | | | | |
| --- | --- | --- | --- | --- | --- | --- | --- | --- | --- | --- | --- | --- |
|  |  | **SS** | | |  | **SPV** | | |  | **SIV** | | |
| **Process** |  | **Enrich-Scorea** |  | **FDR** |  | **Enrich-Scorea** |  | **FDR** |  | **Enrich-Scorea** |  | **FDR** |
| Antioxidant activity |  | 0.00 |  | 1.000 |  | 0.00 |  | 1.000 |  | 2.30 |  | 0.053 |
| Apoptosis regulation |  | 1.18 |  | 0.491 |  | 2.58 |  | 0.024 |  | 1.36 |  | 0.492 |
| Blood vessel development |  | 1.42 |  | 0.224 |  | 2.12 |  | 0.061 |  | 0.00 |  | 1.000 |
| Cell motion |  | 3.18 |  | 6.19 x 10^-3^ |  | 5.56 |  | 3.73 x 10^-5^ |  | 4.15 |  | 2.29 x 10^-4^ |
| Chemotaxis |  | 4.57 |  | 2.09 x 10^-5^ |  | 5.86 |  | 1.08 x 10^-7^ |  | 5.01 |  | 6.25 x 10^-5^ |
| EGF-signalling |  | 2.39 |  | 3.11 x 10^-4^ |  | 2.41 |  | 0.008 |  | 3.88 |  | 1.65 x 10^-4^ |
| Glycosaminoglican binding |  | 2.86 |  | 4.13 x 10^-3^ |  | 2.36 |  | 0.032 |  | 2.31 |  | 0.038 |
| Immune cells activation |  | 1.50 |  | 0.108 |  | 3.30 |  | 0.003 |  | 0.22 |  | 0.999 |
| Immune system development |  | 0.00 |  | 1.000 |  | 2.62 |  | 0.029 |  | 1.03 |  | 0.647 |
| Lung development |  | 0.00 |  | 1.000 |  | 1.27 |  | 0.466 |  | 2.31 |  | 0.070 |
| **Neuron projection morphogenesis** |  | **0.00** |  | **1.000** |  | **0.00** |  | **1.000** |  | **3.68** |  | **1.76 x 10^-3^** |
| Regulation of synaptic transmission |  | 0.00 |  | 1.000 |  | 1.77 |  | 0.059 |  | 0.59 |  | 0.999 |
| Response to microorganisms |  | 4.59 |  | 2.00 x 10^-5^ |  | 10.78 |  | 1.04 x 10^-8^ |  | 1.78 |  | 0.245 |
| TLR signaling pathway |  | 1.34 |  | 0.291 |  | 1.19 |  | 0.538 |  | 0.64 |  | 0.931 |
| Water channel activity |  | 1.82 |  | 0.027 |  | 1.46 |  | 0.794 |  | 2.68 |  | 3.29 x 10^-3^ |
| a Enrichment score. SS: unventilated spontaneously breathing septic animals; SPV: septic animals ventilated with protective mechanical ventilation; SIV: septic animals ventilated with injurious mechanical ventilation; FDR: false discovery rate; TLR: toll-like receptor. | | | | | | | | | | | | |
