# Supplementary material for: A vascular endothelial growth factor receptor gene variant is associated with susceptibility to acute respiratory distress syndrome
Source: Intensive Care Med Exp. 2018 Jul 9;6:16. doi: 10.1186/s40635-018-0181-6 (PMC6037659; doi:10.1186/s40635-018-0181-6)
Supplement: Supplementary file 3 — Table S3. Protein interaction network analysis (data obtained from Acosta-Herrera et al. PLoS One 2015, 10:e0132296). Summary results of the protein-protein interaction network analysis as part of the lung transcriptomic study previously published. (DOC 40 kb) [file 40635_2018_181_MOESM3_ESM.doc]

| **Table S3.** **Protein interaction network analysis (data obtained from Acosta-Herrera et al. PLoS One 2015, 10:e0132296).** | | | | | | | | | | | | |
| --- | --- | --- | --- | --- | --- | --- | --- | --- | --- | --- | --- | --- |
|  |  | SS | | |  | SPV | | |  | SIV | | |
| Annotation (pathway/process) |  | Score^a^ |  | FDR |  | Score^a^ |  | FDR |  | Score^a^ |  | FDR |
| Signaling by VEGF |  | 2.38 |  | 0.009 |  | 2.37 |  | 0.040 |  | 1.53 |  | 0.137 |
| Chemokine receptors bind chemokines |  | -- |  | -- |  | -- |  | -- |  | 1.22 |  | 1.73x10^-5^ |
| Prostanoid hormones |  | -- |  | -- |  | 1.55 |  | 0.329 |  | -- |  | -- |
| Toll like receptor 4 cascade |  | 1.59 |  | 0.001 |  | -- |  | -- |  | 1.56 |  | 0.001 |
| MYD88 cascade |  | 1.35 |  | 0.041 |  | -- |  | -- |  | 1.31 |  | 0.044 |
| P75NTR signal via NFKB |  | -- |  | -- |  | -- |  | -- |  | 1.97 |  | 0.020 |
| Viral DSRNA TLR3 TRIF complex activates RIP1 |  | -- |  | -- |  | -- |  | -- |  | 2.14 |  | 0.019 |
| Human TAK1 activates NFKB by phosphorylation and activation of IKKS complex |  | -- |  | -- |  | -- |  | -- |  | 1.69 |  | 0.026 |
| ^a^Network interconnectivity score. SS: unventilated spontaneously breathing septic animals; SPV: septic animals ventilated with protective mechanical ventilation; SIV: septic animals ventilated with injurious mechanical ventilation; FDR: false discovery rate. | | | | | | | | | | | | |
|
