# Supplementary material for: A vascular endothelial growth factor receptor gene variant is associated with susceptibility to acute respiratory distress syndrome
Source: Intensive Care Med Exp. 2018 Jul 9;6:16. doi: 10.1186/s40635-018-0181-6 (PMC6037659; doi:10.1186/s40635-018-0181-6)
Supplement: Supplementary file 4 — Table S4. Summary results of the transcriptomic study in blood cells from septic and sepsis-derived ARDS patients. Summary results of the validation from GSEA in whole blood performed in a transcriptomic study. (DOC 31 kb) [file 40635_2018_181_MOESM4_ESM.doc]

| **Table S4. Summary results of the transcriptomic study in blood cells from septic and sepsis-derived ARDS patients.** | | | | |
| --- | --- | --- | --- | --- |
| Process |  | Enrich-Score^a^ |  | *p*-value |
| SEMA4D in Semaphorin signaling |  | 0.59 |  | 6.30 x 10^-3^ |
| NETRIN1 signaling |  | 0.59 |  | 6.70 x 10^-3^ |
| Semaphorin interactions |  | 0.52 |  | 5.40 x 10^-3^ |
| ^a^Enrichment score. | | | | |
