# Supplementary material for: A vascular endothelial growth factor receptor gene variant is associated with susceptibility to acute respiratory distress syndrome
Source: Intensive Care Med Exp. 2018 Jul 9;6:16. doi: 10.1186/s40635-018-0181-6 (PMC6037659; doi:10.1186/s40635-018-0181-6)
Supplement: Supplementary file 6 — Table 4. ARDS susceptibility association results in the discovery and replication studies. Summary of association results for the three SNPs associated with ARDS susceptibility in the discovery and replication studies. (DOC 40 kb) [file 40635_2018_181_MOESM6_ESM.doc]

| **Additional file 6: Table 4. ARDS susceptibility association results in the discovery and replication studies.** | | | | | | | | | | | | | | | | | | | | |
| --- | --- | --- | --- | --- | --- | --- | --- | --- | --- | --- | --- | --- | --- | --- | --- | --- | --- | --- | --- | --- |
|  |  |  |  |  |  | Discovery (225 cases:899 controls) | | | | |  | Replication  (661 cases:234 controls) | | | | |  | Meta-analysis (886 cases:1,133 controls) | | |
|  |  |
| SNP |  | Gene |  | Effect/ non-effect allele |  | Allele frequency ^a^ |  | OR (95% CI) |  | *p*-value |  | Allele frequency ^a^ |  | OR (95% CI) |  | *p*-value |  | OR (95% CI) |  | *p*-value |
| rs9513106 |  | *FLT1* |  | C/A |  | 0.44 |  | 0.76  (0.58-0.98) |  | 0.037 |  | 0.31 |  | 0.78 (0.62-0.98) |  | 0.039 |  | 0.77  (0.65-0.92) |  | 0.003 |
| rs11225640 |  | *DYNC2H1* |  | C/T |  | 0.41 |  | 0.88  (0.63-1.24) |  | 0.478 |  | 0.08 |  | 0.79 (0.53-1.18) |  | 0.245 |  | 0.84 (0.65-1.09) |  | 0.195 |
| rs16880534 |  | *ITGA1* |  | G/A |  | 0.35 |  | 1.11  (0.86-1.44) |  | 0.427 |  | 0.22 |  | 1.03 (0.79-1.33) |  | 0.853 |  | 1.07 (0.89-1.28) |  | 0.487 |
| ^a^ Allele frequency was calculated for the effect allele. | | | | | | | | | | | | | | | | | | | | |
